# A Superfolder Green Fluorescent Protein-Based Biosensor Allows Monitoring of Chloride in the Endoplasmic Reticulum

**DOI:** 10.1021/acssensors.2c00626

**Published:** 2022-08-11

**Authors:** Kaavian Shariati, Yaohuan Zhang, Simone Giubbolini, Riccardo Parra, Steven Liang, Austin Edwards, J. Fielding Hejtmancik, Gian Michele Ratto, Daniele Arosio, Gregory Ku

**Affiliations:** † Diabetes Center, University of California San Francisco, San Francisco, California 94143, United States; ‡ Metabolic Biology Graduate Program, Department of Nutritional Science and Toxicity, University of California Berkeley, Berkeley, California 94720, United States; § National Enterprise for NanoScience and NanoTechnology (NEST), Istituto Nanoscienze, Consiglio Nazionale delle Ricerche (CNR) and Scuola Normale Superiore Pisa, Pisa 56127, Italy; ∥ Biological Imaging Development CoLab, University of California San Francisco, San Francisco, California 94143, United States; ⊥ Ophthalmic Genetics and Visual Function Branch, National Eye Institute, Bethesda, Maryland 20892-1860, United States; # CNR, Institute of Biophysics, Via alla Cascata 56/C, Trento 38123, Italy; ∇ CIBIO, University of Trento, Via delle Regole 101, Trento 38123, Italy; ○ Diabetes Center and Department of Medicine, Division of Endocrinology, University of California San Francisco, 513 Parnassus Avenue, Box 0534, San Francisco, California94143, United States

**Keywords:** chloride, endoplasmic reticulum, biosensor, pH, superfolder

## Abstract

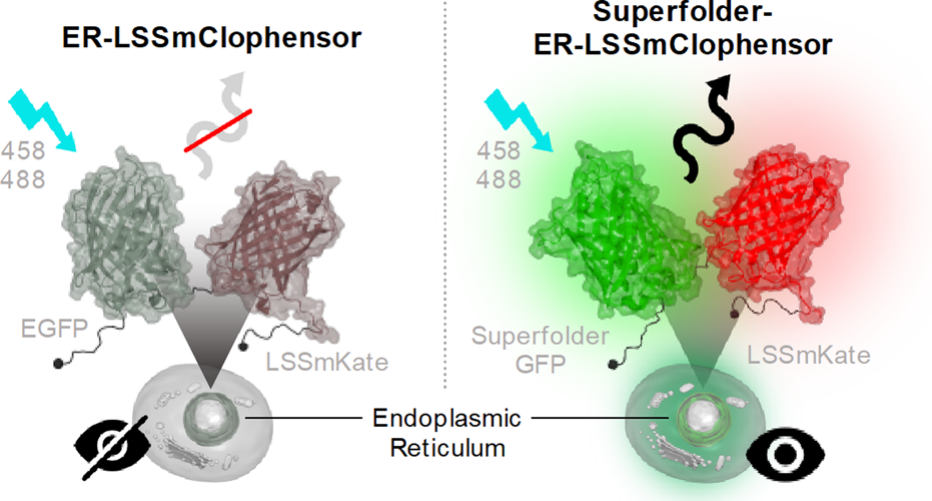

Though the concentration of chloride has been measured in the cytoplasm and in secretory granules of live cells, it cannot be measured within the endoplasmic reticulum (ER) due to poor fluorescence of existing biosensors. We developed a fluorescent biosensor composed of a chloride-sensitive superfolder GFP and long Stokes-shifted mKate2 for simultaneous chloride and pH measurements that retained fluorescence in the ER lumen. Using this sensor, we showed that the chloride concentration in the ER is significantly lower than that in the cytosol. This improved biosensor enables dynamic measurement of chloride in the ER and may be useful in other environments where protein folding is challenging.

Chloride is the most abundant anion in cells, playing a role in diverse cellular processes that range from control of membrane potential and synaptic signaling to cell volume.^1,2^ Disruption of chloride homeostasis occurs in many human diseases. Perhaps, the most well-known chloride-related disease is cystic fibrosis, caused by mutations in the cystic fibrosis transmembrane conductance regulator (CFTR) gene. Mutations in CFTR, which encodes a chloride channel on the plasma membrane, block chloride excretion by airway secretory cells.^3,4^ Furthermore, the role of chloride regulation in brain diseases is a field of growing importance, both from the point of view of pathophysiology and as a target for putative treatments.^5^ Therefore, measuring chloride concentrations in cells is of high relevance to human diseases.

Chemical probes for chloride have largely been replaced by genetically encoded biosensors.^6^ One large class of biosensors was based on a version of a yellow fluorescent protein (YFP) that enhanced chloride binding. One example is Clomeleon, a ratiometric fluorescence resonance energy transfer (FRET)-based chloride sensor, that uses a variant of YFP that is sensitive to chloride.^7^ One disadvantage of YFP-based sensors is that they are strongly influenced by pH so an accurate chloride measurement requires simultaneous measurement of pH. A more recent chloride biosensor, ClopHensor, allows simultaneous measurement of chloride and pH through the Cl and pH sensitivity of E2GFP, a variant of green fluorescent protein (GFP) that is particularly chloride-sensitive.^8^ Having a single biosensor that is also sensitive to pH and chloride allows more accurate determination of chloride in cells.

Chloride is also important in the lumen of intracellular organelles. One key role of chloride channels is the generation of a short circuit current that allows organelle acidification (golgi, endosome, lysosome, and secretory vesicle).^9,10^ Chloride channels have also been implicated in the process of secretion itself.^11^ In the endoplasmic reticulum (ER), chloride movement is proposed to provide an anion countercurrent to allow efficient calcium pumping into the ER by SERCA.^12^ ER chloride may also be important in human disease. A spontaneous mutation in an ER-localized chloride channel, Clcc1, has been found to regulate ER stress and cell death in mice^13^ and a point mutation in CLCC1 causes autosomal-dominant retinitis pigmentosa.^14^

Despite the importance of chloride in the ER, there have been no measurements of chloride within the ER lumen in living cells. To address this gap, we attempted to measure chloride in the ER using large Stokes-shifted mKate2-ClopHensor (LSSmClopHensor^15^) by localizing the sensor in the ER lumen. We found that the ER-localized LSSmClopHensor was very poorly fluorescent, possibly due to the oxidizing environment of the ER.^16^ To overcome this technical limitation of LSSmClopHensor, we created a superfolder GFP-based LSSmClopHensor that is capable of chloride sensing in the unique environment of the ER.

## Experimental Section

### Cells

293T and GL261 cells were cultured in Dulbecco’s modified Eagle medium (DMEM) high glucose (Gibco) with 10% fetal bovine serum, penicillin, and streptomycin (Gibco). To express the biosensor in 293T cells, transient transfection with the jetPRIME (Polyplus) was performed 1–2 days prior to the analysis. MIN6 cells, a generous gift from Dr. Miyazaki,^19^ were grown in DMEM high glucose with pyruvate (Gibco) with 15% fetal bovine serum, penicillin, and streptomycin (Gibco) and 50 μM beta-mercaptoethanol (Sigma). To express the biosensor in MIN6 cells, lentiviral infection was performed at a multiplicity of infection of 0.5 and the analysis was performed within 1 week of infection.

### Molecular Biology

CAG-LSSmClopHensor was previously described.^15^ ER-LSSmClopHensor was cloned into pcdna3 (Invitrogen) by the addition of a KDEL sequence at the C-terminus and a calreticulin leader sequence (MLLSVPLLLGLLGLAVAAPVAT) at the N-terminus. Cytosolic LSSmsfClopHensor was generated by replacing the E2GFP of LSSmClopHensor with superfolder GFP with a T203Y substitution. ER-LSSmsfClopHensor was made by replacement of E2GFP in ER-LSSmClopHensor with superfolder GFP T203Y. The DNA sequences are listed in Supplemental Figure 1. For expression in MIN6 cells, ER-LSSmsfClopHensor or LSSmsfClopHensor was cloned into a lentiviral construct driven by the proximal 362 bases of the insulin promoter.^17^ For bacterial expression, cytosolic LSSmsfClopHensor was cloned into pET21 (Novagen) in frame with a C-terminal His tag. mCherry-Sec61b-C-18 and mCherry-calreticulin-N-16 were gifts from Dr. Michael Davidson. All constructs were confirmed by Sanger sequencing.

### Colocalization

293T cells were transiently transfected with ER-LSSmsfClopHensor and mCherry-Sec61 or mCherry-calreticulin. Forty-eight hours after transfection, Hoechst 33342 was added to a final concentration of 10 μg/mL and cells were imaged sequentially with 488 nm excitation and 500–550 nm emission (ClopHensor), 594 nm excitation and 610–650 nm emission (mCherry), and 405 nm excitation and 450–500 nm emission (Hoechst). For colocalization, Pearson correlation coefficients were calculated for at least 15 cells per construct using CoLoc2 (Fiji).

### Recombinant Protein Expression and Analysis

Proteins were produced in BL21 cells and purified on a nickel column as previously described.^15^ Fluorescence was measured using a SpectraMax M5e (Molecular Devices) using black 96-well plates.

### Two-Photon Microscopy

GL261 cells were imaged at 24 °C using a Bruker 2-photon microscope equipped with a Chameleon Ultra II infrared laser. The power delivered on the sample was carefully matched between the different excitation wavelengths, and the spectra have been corrected for the number of photons on the sample. The details of the quantitative analysis are reported elsewhere.^18^ GL261 cells were incubated with the calibration buffers. Two different solutions (Solution A, 0 mM Cl^–^ and Solution B, 138 mM Cl^–^) were prepared and mixed to obtain different calibration solutions. Solution A was prepared with 20 mM HEPES, 0.6 mM MgSO_4_, 38 mM sodium gluconate, and 100 mM potassium gluconate. Solution B was prepared with 20 mM HEPES, 0.6 mM MgSO_4_, 38 mM NaCl, and 100 mM KCl. The calibration solutions (0, 10, 20, 40, and 80 mM [Cl^–^]) were prepared by mixing Solution A and Solution B in different proportions according to the final desired chloride concentration. Each calibration solution was also supplemented with 5 μM K^+^/H^+^ exchanger nigericin, 5 μM protonophore carbonyl cyanide *p*-chlorophenylhydrazone (CCCP), 5 μM K^+^ ionophore valinomycin, and 10 μM Cl^–^/OH^–^ exchanger tributyltin chloride (TBTC) to equilibrate extra and intracellular ion concentrations. The calibration solutions were adjusted to different pH values (6, 6.3, and 8) with 1 M NaOH. Before the incubation with calibration solutions, the cells were washed twice with the same solution to equilibrate pH and ion concentrations.

### One-Photon Microscopy

Cells were imaged using a Leica SP5 with an incubation chamber set at 37 °C using a 40× oil objective. The pinhole was adjusted to the diameter of the Airy disk. A single confocal slice was obtained after excitation at 458 nm with acquisition at 500–550 nm, 580–640 nm, and transmitted light. A second excitation was performed at 488 nm with acquisitions in the same channels. For live cell measurements of chloride and pH, the cells were imaged in Hank’s balanced salt solution with calcium and magnesium (Gibco 14025-076) and 50 mM HEPES, pH 7.4. For the in-cell calibration, prior to imaging, the cells were washed with 20 mM HEPES, 0.6 mM MgSO_4_, 38 mM sodium gluconate, 100 mM potassium gluconate, 5 μM nigericin, 5 μM CCCP, 5 μM valinomycin, and 10 μM TBTC. The chloride anion replaced the gluconate anion to achieve the correct final chloride concentration. The pH was adjusted with sodium hydroxide. Five washes were performed with 2 min between washes and the cells were imaged immediately. To calculate the chloride and pH values, each image (containing ∼50–100 cells) was masked based on the fluorescence above background. Bleed-through from sfGFP or from LSSmKate2 was subtracted using images from cells expressing only sfGFP T203Y or LSSmKate2 as previously described.^14^ For time-lapse imaging, the buffer was changed from 0 mM [Cl^–^] to 90 mM [Cl^–^] after acquisition of the first image (marked as time 0) and images were taken every 12 s. For measurement of stability during photobleaching, ER-targeted sfGFP T203Y or ER-targeted LSSmKate2 was transfected into 293T cells as above and imaged every second for 175 s using the 488 nm laser line delivering 77.6 μW.

### Estimation of Chloride

Chloride was estimated from *R*_Cl_ = ratio of cyan (excitation at 458 nm and emission at 500–550 nm) to red (excitation at 458 nm and emission at 580–640 nm). The pH was estimated from *R*_pH_ = ratio of green (excitation at 488 nm and emission at 500–550 nm) to cyan (excitation at 458 nm and emission at 500–550 nm) as has been previously described for LSSmClopHensor^14^ with the following modifications. To control the fluctuations in illumination of the 458 and 488 lasers during imaging and calculation of *R*_pH_, the cyan and green channels were normalized to the transmitted light collected from that same image. For the estimation of pH, the values of *R*_A_, *R*_B_, and p*K*_a_ were calculated based on the in-cell calibration using the Levenberg–Marquardt algorithm as implemented in lmfit 1.0.3 (nonlinear least-squares minimization and curve fitting for Python). The dissociation constant of chloride (*K*_d_Cl) was calculated at that pH based on an exponential fit between pH and *K*_d_Cl (Supplemental Figure 2B). The *K*_d_Cl at each pH was experimentally determined using the recombinant protein with a best fit of [Cl^–^] versus *R*_Cl_ using lmfit with the equation *R*_Cl_ = (*R*_0_ + *R*_1_ × [Cl^–^]/*K*_d_Cl)/(1 + ([Cl^–^]/*K*_d_Cl)) where *R*_1_ is the R_Cl_ at infinite [Cl^–^], *R*_0_ is the *R*_Cl_ at zero [Cl^–^], and *K*_d_Cl is the *K*_d_ for [Cl^–^] at that pH (Supplemental Figure 2A). From the in-cell calibration, we noted that the *R*_Cl_ values at zero [Cl^–^] (*R*_0Cl_) were modestly pH-dependent, perhaps due to the slight pH sensitivity of LSSmsfClopHensor at 458 nm excitation and 500–550 nm emission (Figure 2B, Supplemental Figure 2A). Therefore, a linear fit based on the measured pH was used to determine *R*_Cl_ at zero [Cl^–^] for each sample based on the in-cell calibration (Supplemental Figure 3).

## Results and Discussion

**Figure 1:**
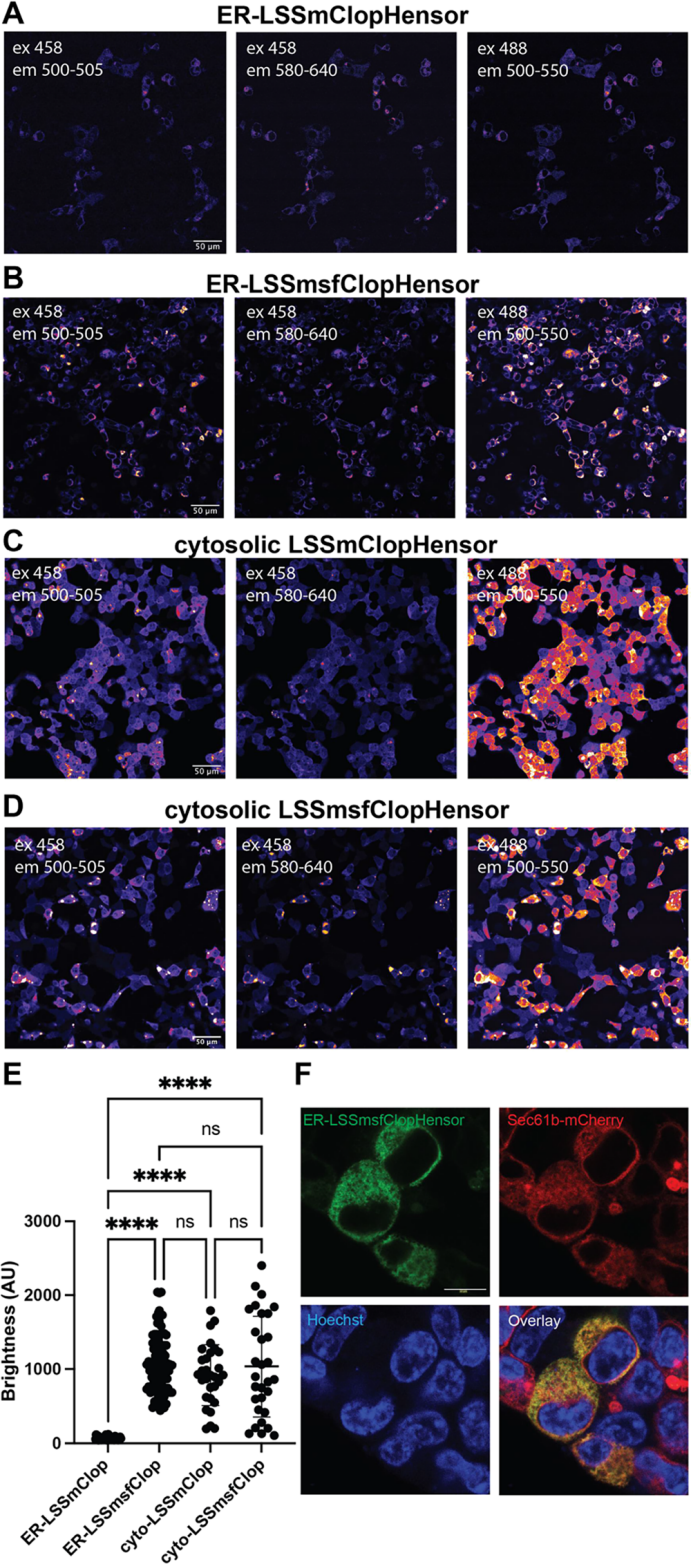
Increased brightness of ER-targeted LSSmsfClopHensor. (A) 293T cells expressing ER-targeted LSSmClopHensor, (B) ER-targeted superfolder LSSmClopHensor, (C) cytosolic LSSmClopHensor, and (D) cytosolic LSSmsfClopHensor. (E) Intensity per cell of the indicated ClopHensor with 488 nm excitation and 500–550 nm emission, *n* = 30–100 cells per group, ****p < 0.0001 (one-way ANOVA and Tukey’s post-hoc test). For panels (A–E), identical instrument settings were used. (F) 293T cells were cotransfected with ER-LSSmsfClopHensor and mCherry-Sec61 and imaged for GFP, mCherry, and Hoechst.

To monitor the concentration of chloride in the lumen of the ER, we modified LSSmClopHensor, a ratiometric chloride and pH sensor that utilizes the chloride and pH sensitivity of E2GFP and a large Stokes-shifted mKate2.^15^ We placed an amino-terminal calreticulin leader sequence and a C-terminal KDEL retention signal to localize LSSmClopHensor to the ER. Such a strategy has been successfully used for monitoring the calcium concentration in the ER using a calcium biosensor.^19^ Unfortunately, E2GFP fluorescence after excitation at 488 or 458 nm was nearly undetectable (Figure 1A). One possibility is that E2GFP is glycosylated in the ER, rendering it nonfluorescent. However, we could not computationally identify a consensus *N*-linked glycylation site in E2GFP. Instead, we speculated that the loss of E2GFP fluorescence could be due to the formation of disulfide-linked oligomers in the environment of the ER. The closely related EGFP misfolds in the ER due to the disulfides formed at cysteines 49 and 71^16^ and the E2GFP retains both cysteines. The superfolder GFP has been shown to be relatively resistant to oxidizing environments such as the ER.^20^ A superfolder GFP version of pHluorin has been generated to measure the pH in the ER of yeast.^21^ Therefore, we replaced E2GFP with superfolder GFP and introduced T203Y substitution to attempt to confer increased chloride sensitivity.^22^ We termed this biosensor **L**arge **S**tokes-**S**hifted **m**Kate-**s**uper**f**older ClopHensor (LSSmsfClopHensor). When transfected into cells, ER-LSSmsfClopHensor was substantially brighter than the original ER-LSSmClopHensor (Figure 1B). When LSSmsfClopHensor was expressed in the cytosol (Figure 1D), it was comparable in brightness to the original LSSmClopHensor (Figure 1C,E). Importantly, ER-LSSmsfClopHensor colocalized with the ER markers Sec61 (Figure 1F) and calreticulin (Supplemental Figure 4A). The Pearson correlation coefficient average was 0.716 for Sec61 and 0.805 for calreticulin (*p* < 0.0001 compared to an expected value of 0 correlation by a one-sample Student’s *t*-test) (Supplemental Figure 4B).

**Figure 2:**
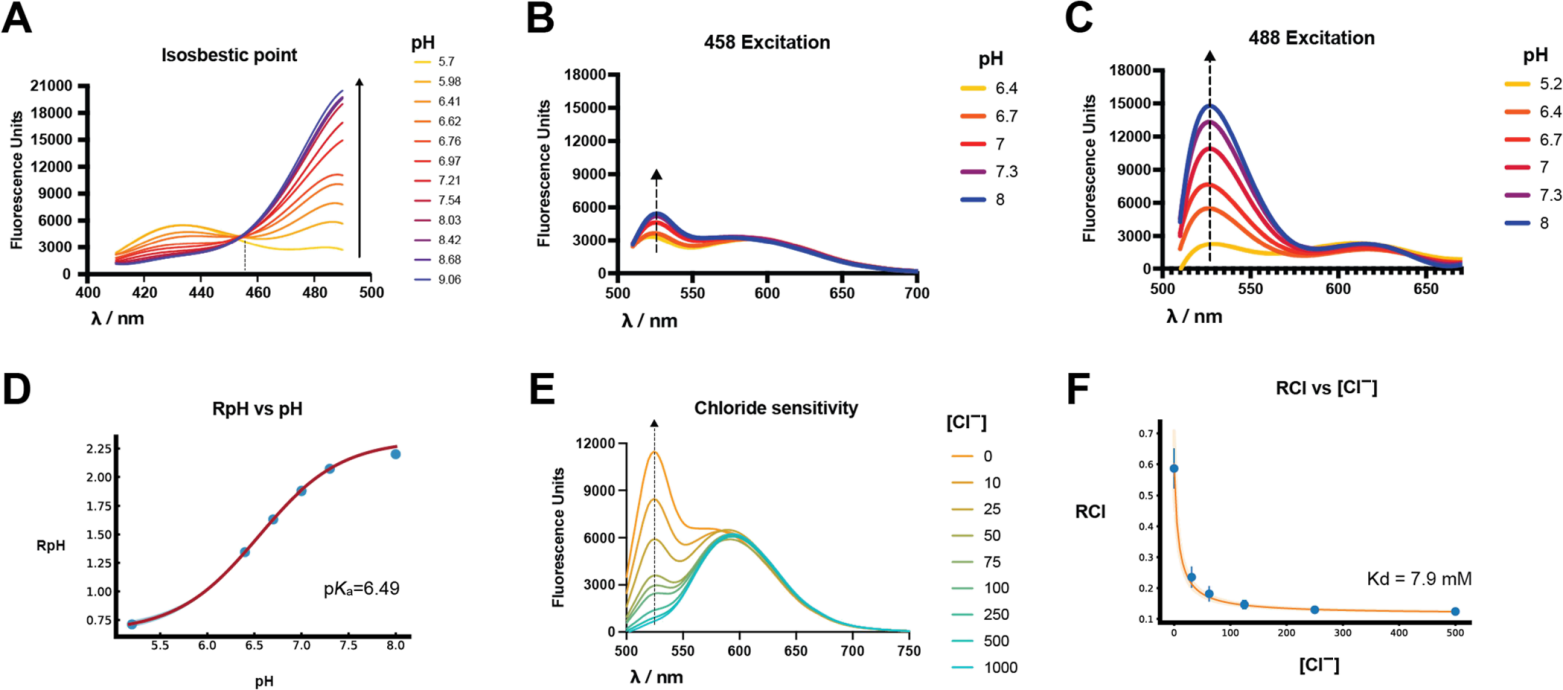
LSSmsfClopHensor retains sensitivity to [Cl^–^] and pH. (A) Excitation spectra at indicated pH of recombinant LSSmsfClopHensor with emission measured at 520 nm. The isosbestic point is shown at 455 nm. (B) Emission spectra at the indicated pH after excitation at 458 nm. (C) Emission spectra at the indicated pH after excitation at 488 nm. (D) *R*_pH_ (ratio of emission at 500–550 nm between 488 nm excitation and 458 nm excitation) over different pH conditions. (E) Emission curves after excitation at 458 nm over indicated [Cl^–^]. (F) *R*_Cl_ (emission at 500–550 nm divided by emission at 580–640 nm with 458 nm excitation) over indicated [Cl^–^] at pH 6.5, 37 °C. *n* = 3. For panels (B, C, and E), the arrow indicates the peak of GFP emission.

To validate that ER-LSSmsfClopHensor retains the ability to detect [Cl^–^] and pH, we expressed LSSmsfClopHensor in bacteria. We found that purified LSSmsfClopHensor had an isosbestic point of 455 nm, close to that of the original LSSmClopHensor (Figure 2A). Furthermore, emission at 500–550 nm after excitation at 488 nm (green) retained sensitivity to pH (Figure 2C), while emission at 500–550 nm after 458 nm excitation (cyan) was substantially less responsive to pH (Figure 2B), similar to the original LSSmClopHensor.^15^*R*_pH_, the ratio of green to cyan, demonstrated the predicted relationship with pH^15^ (Figure 2D). Curve fitting showed an *R*_A_ of 0.62 with a 95% confidence interval of (0.57–0.67) and an *R*_B_ of 2.27 (2.23–2.31) with a calculated p*K*_a_ of 6.49 (6.43–6.55). As expected, p*K*_a_ was dependent on temperature (Supplemental Figure 5).

Emission at 500–550 after excitation at 458 nm (cyan) was sensitive to [Cl^–^], while emission at 580–640 nm after excitation at 458 nm (red, from LSSmKate2) was not (Figure 2E). *R*_Cl_ (cyan divided by red) plotted versus [Cl^–^] showed that the best fit-determined *K*_d_ for [Cl^–^] of LSSmsfClopHensor was 7.9 mM (4.1–11.7) at pH 6.4, 37 °C (Figure 2F). The *K*_d_Cl varied slightly with temperature but not in a simple fashion (Supplemental Figure 6). These data show that LSSmsfClopHensor should be calibrated at the same temperature as that used to make measurements.

Two-photon microscopy is a valuable tool for quantitative pH and [Cl^–^] imaging in vivo,^18^ and the repertoire of available genetically encoded sensors is continuously expanding;^23^ therefore, we analyzed the behavior of this new sensor under two-photon excitation.

**Figure 3:**
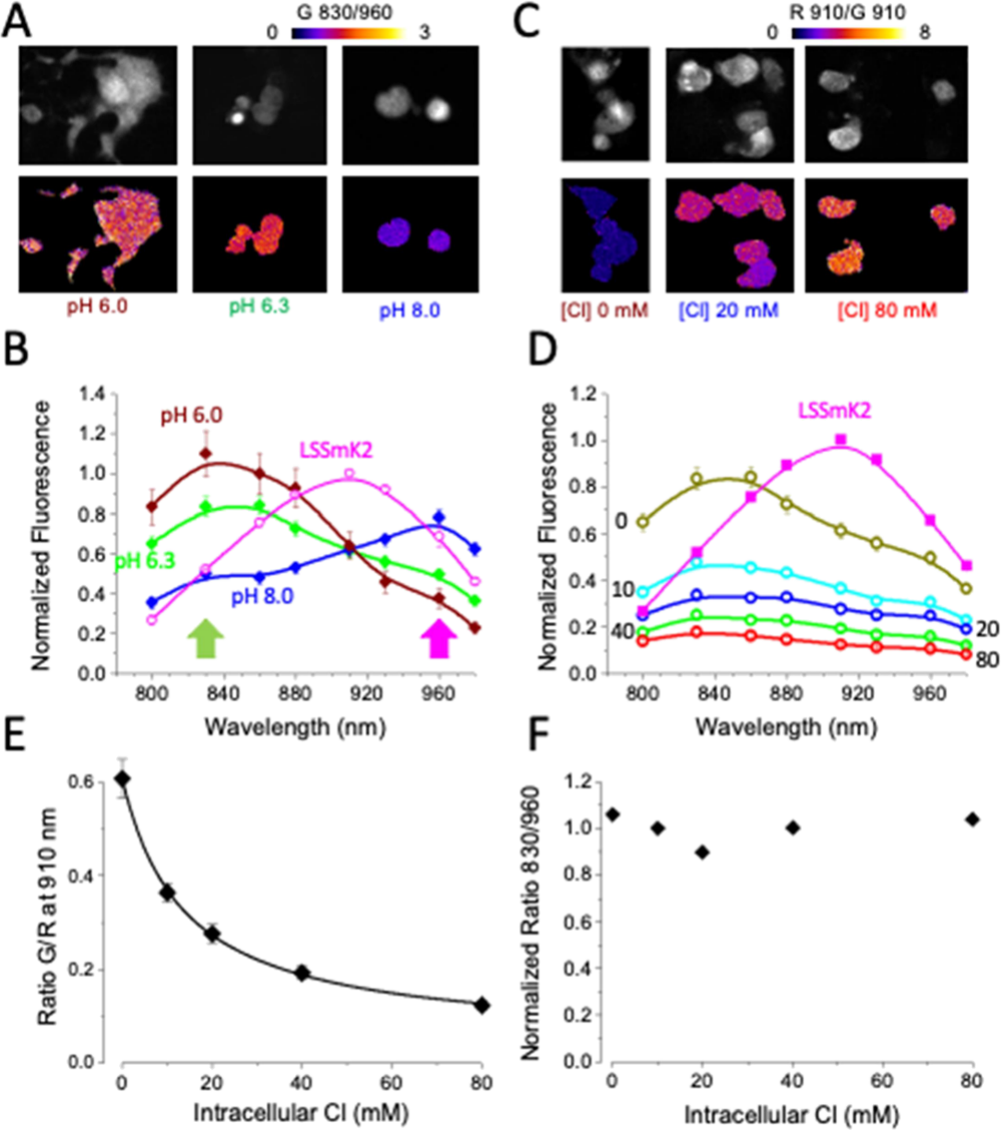
Two-photon spectra of sfClopHensor. (A) Images showing the shift of the 2-photon excitation spectra between the protonated and nonprotonated states. The upper panels show the fluorescence averaged on all excitation wavelengths, while the lower panels show the ratio of the fluorescence in the green channel excited at 830 nm divided by G960. (B) Excitation peak of sfClopHensor at the three indicated pH values. The magenta data points indicate the spectrum of LSSmKate2 that is independent on pH or [Cl^–^]. The arrows indicate the peaks of the sensor in the two protonation states. The isosbestic point is found to be at about 910 nm, as for LSSmClopHensor. All cells were imaged in zero [Cl^–^] in the presence of the ionophore cocktail. The line fitting the data at pH 6.3 has been obtained by computing the linear combination of the spectra measured at pH 6.0 and 8.0.^18^ (C) Quenching of green fluorescence at increasing [Cl^–^] concentrations. The lower images show the ratio of the fluorescence in the red and green channels at the isosbestic point (910 nm). (D) Dependency of sfGFP fluorescence on intracellular [Cl^–^]. The spectra have been obtained at the values of intracellular [Cl^–^] indicated by the labels at pH 6.3. (E) Calibration of the G/R ratio on intracellular [Cl^–^]. The line is provided by the fit of eq 1. (F) Ratio of the fluorescence in the G channel measured at the peak of the protonated (830 nm) and deprotonated excitation spectra (960 nm). This ratio does not show a clear dependency on [Cl^–^] since the sensor affinity to H^+^ is independent of intracellular [Cl^–^].

We transfected cytosolic LSSmsfClopHensor into GL261 cells and measured the excitation spectra in the presence of ionophores at three different pH values (Figure 3A,B). As for LSSmClopHensor, we observed two excitation peaks at 830 and 960 nm for the protonated and unprotonated sensor, respectively. The isosbestic point was at 910 nm. The pH can be computed by fitting the spectra with a linear combination of the spectra at pH 6 ad 8 as detailed elsewhere.^18^ The dependency of green fluorescence on chloride concentration at pH 6.3 is shown in Figure 3C,D.

The quenching of the fluorescence in the green channel is shown in Figure 3E. The data points have been fitted with the function:
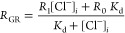
1where *R*_GR_ is the ratio observed at the different values of [Cl^–^]_*i*_. The fit of these data returns a *K*_d_ for [Cl^–^] of ∼14 mM at pH 6.3 at 24 °C. We note that *R*_pH_ was not dependent on [Cl^–^] (Figure 3F).

**Figure 4:**
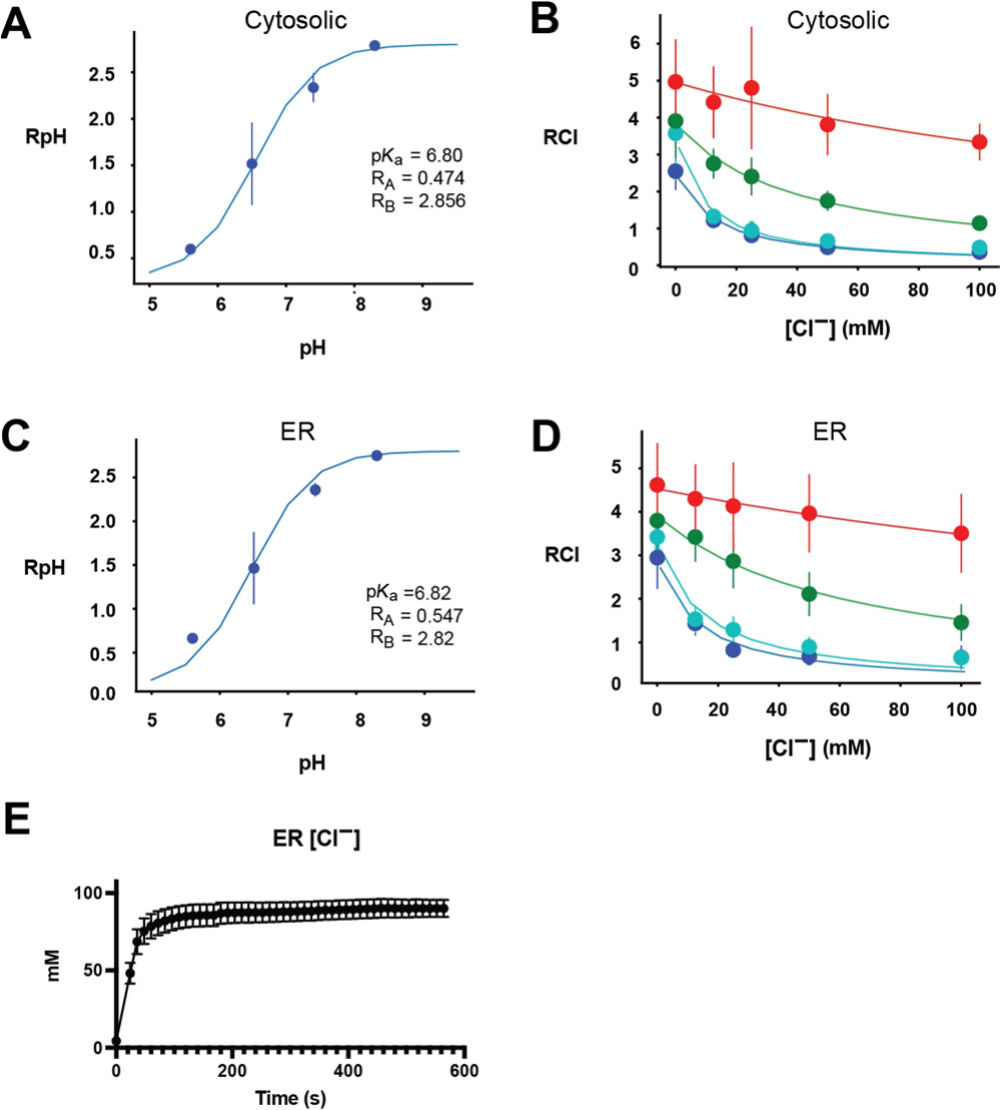
In-cell calibration of LSSmsfClopHensor. (A) *R*_pH_ for cells at the indicated pH values for cytosolic LSSmsfClopHensor. (B) *R*_Cl_ for cells at the indicated pH and chloride values for cytosolic LSSmsfClopHensor. (C) As in panel (A) but for ER LSSmsfClopHensor. (D) As in panel (B) but for ER LSSmsfClopHensor. For panels (B and D), red line = pH 8.3, green line = pH 7.4, cyan line = pH 6.5, and blue line = pH 5.6. Error bars indicate standard deviation. *N* = 4 for each condition. (E) As in panels (A–D), but cells were washed in a buffer containing 0 mM [Cl^–^], pH 7.3 and changed to 90 mM [Cl^–^], pH 7.3 after the first image at time 0. *n* = 7 independent fields. Chloride was calculated as described in the methods with the pH set to 7.3. Error bars show the standard error.

We then performed calibration of cytosolic and ER-LSSmsfClopHensor in 293T cells using single-photon microscopy again by clamping pH and [Cl^–^] in the presence of ionophores to allow equilibrium between the ER/cytosol and the extracellular space. As expected, *R*_pH_ varied with pH in the cytosol (Figure 4A) and in the ER (Figure 4C). *R*_Cl_ was dependent on pH and [Cl^–^] (Figure 4B,D). For *R*_Cl_, the ER and cytosolic calibration curves were not statistically significantly different (three-way ANOVA: *F* = 2.76, *p* = 0.09). On the other hand, as expected, [Cl^–^] and pH had a significant effect on *R*_Cl_ (*F* = 88.62, *p* < 2 × 10^–16^ and *F* = 266.821, *p* < 2 × 10^–16^, respectively) and pH and [Cl^–^] interacted significantly (F = 4.566, *p* = 1.64 × 10^–6^). Therefore, the cellular compartment did not have a significant effect on *R*_Cl_. However, to minimize the artifact when comparing different compartments, we calculated [Cl^–^] based on the in-cell calibration from that compartment. We determined *R*_A_ = 0.47 with a 95% confidence interval of (0.46–0.49), *R*_B_ = 2.86 (2.83–2.88), and p*K*_a_ = 6.80 (6.76–6.84) for cytosolic LSSmsfClopHensor and *R*_A_ = 0.55 (0.53–0.56), *R*_B_ = 2.82 (2.80–2.85), and a p*K*_a_ of 6.82 (6.77–6.89) for the ER-localized sensor. We noted that the *R*_Cl_ at zero chloride was modestly pH-dependent, possibly from the small pH sensitivity of LSSmsfClopHensor emission at 500–550 nm after 458 nm excitation (Figure 2B); we compensated for this by adjusting *R*_0Cl_ at different pH values (see the Experimental Section).

We measured the photobleaching of each component of LSSmsfClopHensor to evaluate LSSsfClopHensor’s fidelity during dynamic imaging. ER-localized sfGFP T203Y was considerably more stable to photobleaching than ER-localized LSSmKate2 (Supplemental Figure 7) at physiological [Cl^–^] in 293T cells. The apparent photostability of sfGFP T203Y will be increased in the presence of chloride as this renders a fraction of sfGFP nonfluorescent and therefore stable to photobleaching. This difference in photobleaching between LSSmKate2 and sfGFP could result in the artifactual reduced estimated [Cl^–^] concentration after repeated high-power excitation.

To show the ability of LSSmsfClopHensor to dynamically measure changes in [Cl^–^], we measured ER [Cl^–^] in live cells after acutely changing the extracellular [Cl^–^] from 0 to 90 mM in the presence of ionophores as was done in the in-cell calibration. The measured [Cl^–^] approached 90 mM within 40 s (Figure 4E). Notably, since the laser power was <20% of that used in the photobleaching protocol, we did not see a reduction in estimated [Cl^–^] over time.

**Figure 5:**
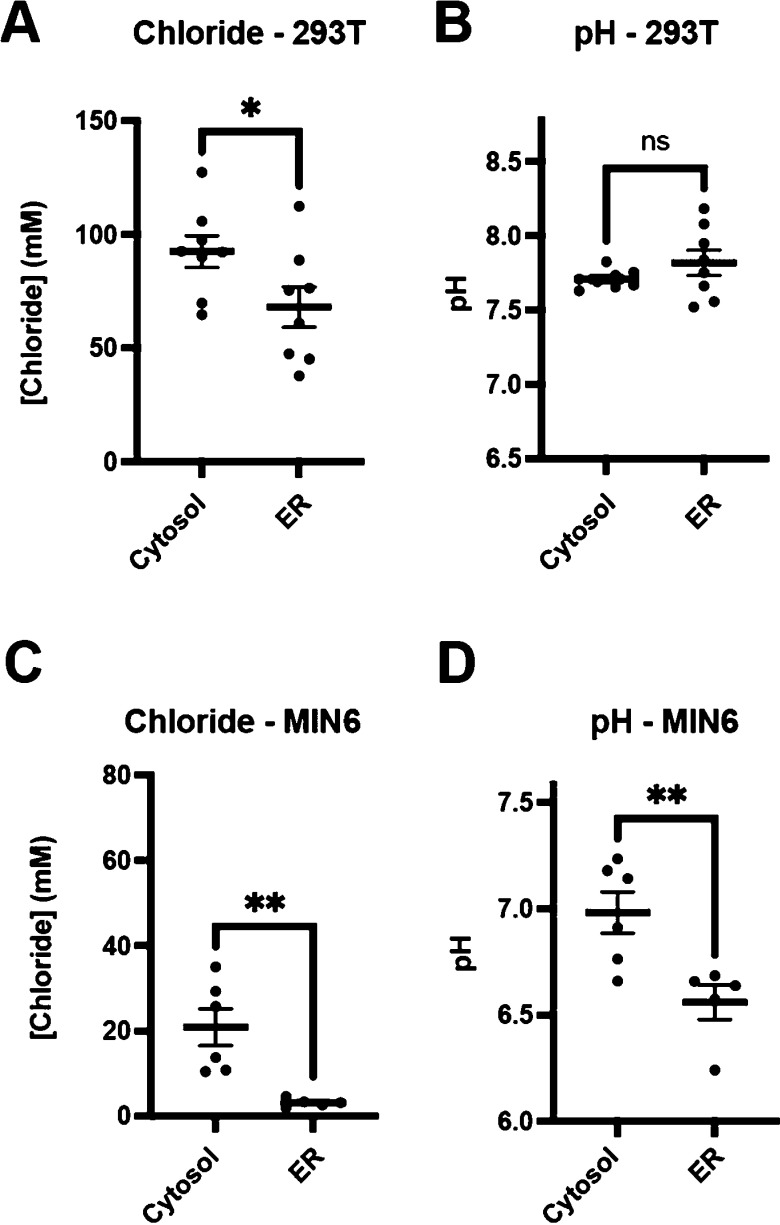
ER chloride concentrations are lower than cytosolic chloride concentrations. (A) [Cl^–^] in the cytosol or in the ER in 293T cells. (B) pH in the cytosol versus the ER in 293T cells. (C) [Cl^–^] in the cytosol or the ER in MIN6 cells. (D) pH in the cytosol or the ER in MIN6 cells. The horizontal line indicates the mean and bars indicate the standard error. *N* = 8 independent fields of cells for panels (A and B) and *N* = 5 (cytosol) or 6 (ER) for panels (C and D). *p < 0.05 and **p < 0.01 by Welch’s *t*-test.

Having validated ER-LSSmsfClopHensor, we measured ER [Cl^–^] in unperturbed cells using one-photon microscopy. In 293T cells, we found that the [Cl^–^] in the cytosol was 92.5 mM using cytosolic LSSmsfClopHensor. In contrast, the [Cl^–^] in the ER lumen was significantly lower at 68.0 mM (*p* < 0.05 vs cytosolic [Cl^–^]) (Figure 5A). The pH in the ER was not significantly different between the ER and the cytosol (Figure 5B). To confirm this difference between ER [Cl^–^] and cytosolic [Cl^–^], we measured [Cl^–^] in the mouse MIN6 pancreatic beta cell line. Though the cytosolic [Cl^–^] was lower than that of 293T cells, we again found that the [Cl^–^] in the ER was significantly lower than that measured in the cytosol (3.16 mM vs 20.9 mM, *p* < 0.01, Figure 5C). The cytosolic pH in MIN6 cells was 6.98, which is inline with the previous measurements of cytosolic pH in beta cells.^24^ We found that the pH in the ER (6.56) was significantly lower than that in the cytosol in MIN6 cells (*p* < 0.01, Figure 5D). The difference in pH between the cytosol and the ER in MIN6 cells highlights a key advantage of LSSmsfClopHensor over FRET-based chloride biosensors, which are not internally corrected for pH. If the pH was assumed to be 7.4 for all cells and compartments, [Cl^–^] would have been overestimated in MIN6 cells and underestimated in 293T cells in both compartments. Nonetheless, even without pH correction, there was a significant decrease in [Cl^–^] in the ER of both MIN6 and 293T cells compared to that in the cytosol, showing that the reduction in chloride in the ER compared to the cytosol is not a measurement artifact of a difference in pH (Supplemental Figure 8). Finally, we measured ER [Cl^–^] in 293T cells after stimulation with the muscarinic agonist carbachol, which reduces the ER calcium levels.^25^ We found that carbachol did not alter ER [Cl^–^] or pH (Supplemental Figure 9A). Treatment of 293T cells with the SERCA inhibitor thapsigargin also did not change ER [Cl^–^] (Supplemental Figure 9B). However, we cannot rule out a small change in [Cl^–^] that might be expected if ER chloride decreases at the same magnitude as ER calcium does (<1 mM).^26^

Our data show that ER [Cl^–^] is significantly lower than that in the cytosol, a new observation made possible by this improved biosensor. The biological significance of this difference is not yet known, but measurement of ER [Cl^–^] is an important first step toward understanding the role of this anion in ER biology.

## Conclusions

We describe an improved biosensor for chloride and pH measurements in live cells. LSSmsfClopHensor allows measurement of ER [Cl^–^] using single- or dual-photon microscopy and its improved folding properties may make it useful for the detection of chloride and pH in other cellular compartments where protein folding is challenging.

## Supporting Information Available

DNA sequences of LSSmsfClopHensor and ER-LSSmsfClopHensor; a plot of the dissociation constant of chloride as a function of pH of recombinant LSSmsfClopHensor; a plot of *R*_Cl_ versus pH from the in-cell calibration; colocalization of ER-LSSmsfClopHensor with calreticulin; temperature dependence of *R*_pH_; temperature dependence of *R*_Cl_; non-pH adjusted measurements of [Cl^–^]; and dynamic measurement of [Cl^–^] after carbachol or thapsigargin treatment (PDF)
