# Supplementary material for: A Superfolder Green Fluorescent Protein-Based Biosensor Allows Monitoring of Chloride in the Endoplasmic Reticulum
Source: ACS Sens. 2022 Aug 11;7(8):2218–24. doi: 10.1021/acssensors.2c00626 (PMC9425558; doi:10.1021/acssensors.2c00626)
Supplement: Supplementary file 1 — se2c00626_si_001.pdf [file se2c00626_si_001.pdf]

Supplemental Data:

A superfolder GFP-based biosensor allows monitoring of chloride in the endoplasmic reticulum

Kaavian Shariati‡, Yaohuan Zhang‡, Simone Giubbolini, Riccardo Parra, Steven Liang, Austin Edwards, J. Fielding Hejtmancik, Gian Michele Ratto, Daniele Arosio, Gregory Ku*

Kaavian Shariati -- Diabetes Center, University of California San Francisco, San Francisco, CA, 94143, USA.
Yaohuan Zhang -- Metabolic Biology Graduate Program, Department of Nutritional Science and Toxicity, University of California Berkeley, Berkeley, CA, 94720.
Simone Giubbolini -- National Enterprise for nanoScience and nanoTechnology (NEST), Istituto Nanoscienze, Consiglio Nazionale delle Ricerche (CNR) and Scuola Normale Superiore Pisa, 56127 Pisa, Italy.
Riccardo Parra -- National Enterprise for nanoScience and nanoTechnology (NEST), Istituto Nanoscienze, Consiglio Nazionale delle Ricerche (CNR) and Scuola Normale Superiore Pisa, 56127 Pisa, Italy.
Steven Liang -- Diabetes Center, University of California San Francisco, San Francisco, CA, 94143, USA.
Austin Edwards – Biological Imaging Development CoLab, University of California San Francisco, San Francisco, CA, 94143, USA.
J. Fielding Hejtmancik -- Ophthalmic Genetics and Visual Function Branch, National Eye Institute, Bethesda, MD, 20892, USA.
Gian Michele Ratto -- National Enterprise for nanoScience and nanoTechnology (NEST), Istituto Nanoscienze, Consiglio Nazionale delle Ricerche (CNR) and Scuola Normale Superiore Pisa, 56127 Pisa, Italy.
Daniele Arosio -- Institute of Biophysics, CNR, Via alla Cascata 56/C, 38123 Trento, Italy, CIBIO, University of Trento, Via delle Regole 101, 38123 Trento, Italy.

Supplemental Figure 1: Nucleotide sequences of ER-LSSmsfClopHensor and LSS msfClopHensor. Underline indicates the start codon. Cyan indicates the ER targeting sequences. Green indicates superfolder GFP. Red indicates LSSmKate2.

Nucleotide sequence of ER-LSSmsfClopHensor:

CACCATGCTGCTGCCCGTCCCCCTGCTGCTGGGCCTGCTGGGCGCCGCCGCCGA
CGTGAGCAAGGGCGAGGAGCTGTTCACCGGGGTGGTGCCCATCCTGGTCGAGCT
GGACGGCGACGTAAACGGCCACAAGTTCAGCGTGTCCGGCGAGGGCGAGGGCG
ATGCCACCTACGGCAAGCTGACCCTGAAGTTCATCTGCACCACCGGCAAGCTGCC
CGTGCCCTGGCCCACCCTCGTGACCACCCTGACCTACGGCGTGCAGTGCTTCAGC
CGCTACCCCGACCACATGAAGCAGCACGACTTCTTCAAGTCCGCCATGCCCGAAG
GCTACGTCCAGGAGCGCACCATCTTCTTCAAGGACGACGGCAACTACAAGACCCG
CGCCGAGGTGAAGTTCGAGGGCGACACCCTGGTGAACCGCATCGAGCTGAAGGG
CATCGACTTCAAGGAGGACGGCAACATCCTGGGGCACAAGCTGGAGTACAACTAC
AACAGCCACAACGTCTATATCATGGCCGACAAGCAGAAGAACGGCATCAAGGTGA
ACTTCAAGATCCGcCACAACATCGAGGACGGCAGCGTGCAGCTCGCCGACCACTA
CCAGCAGAACATCCCCATCGGCGACGGCCCcGTGCTGCTGCCCGACAACCACTAC
CTGAGCTACCAGTCCGCCCTGAGCAAAGACCCCAACGAGAAGCGCGATCACATGG
TCCTGCTGGAGTTCGTGACCGccGCCGGGATCACTCTCGGCATGGACGAGCTGTA
CAAGCGCGGATCCGCGTCTGGTGGTGGTGGTGGTCTAGTTCCACGTGGATCTGCC
TCAGGAGCAGTGAGCGAGCTGATTAAGGAGAACATGCACATGAAGCTGTACATGG
AAGGCACCGTGAACAACCACCACTTCAAGTGCACATCCGAGGGCGAAGGCAAGCC
CTACGAGGGCACCCAGACCATGAGAATCAAGGTGGTCGAGGGCGGCCCTCTACC
CTTCGCCTTCGACATCTTGGCTACCAGCTTCATGTACGGCAGCTACACCTTCATCA
ACCACACCCAGGGCATCCCCGACTTCTTTAAGCAGTCCTTCCCTGAGGGCTTCACA
TGGGAGAGAGTCACCACATACGAAGACGGGGGCGTGCTGACCGCTACCCAGGAC
ACCAGCCTCCAGGACGGTTGCCTCATCTACAACGTCAAGATCAGAGGGGTGAACT
TCACATCCAACGGCCCTGTGATGCAGAAGAAAACACTCGGCTGGGAGGCCGGCA
CCGAGATGCTGTACCCCGCTGACGGCGGCCTGGAAGGCAGATCTGACGACGCCC
TGAAGCTCGTGGGCGGGGGCCACCTGATCTGCAACTTGAAGAGCACATACAGATC
CAAGAAACCCGCTAAGAATCTCAAGGTGCCCGGCGTCTACTATGTGGACCGAAGA
CTGGAAAGAATCAAGGAGGCCGACAAAGAGACCTACGTCGAGCAGCACGAGGTG
GCTGTGGCCAGATACTGCGACCTCCCTAGCAAACTGGGGCACCGCGGCAAGGAC
GAGCTGTAA

Amino acid sequence of ER-LSSmsfClopHensor:

MLLPVPLLLGLLGAAADVSKGEELFTGVVPILVELDGDVNGHKFSVSGEGEGDATYGK
LTLKFICTTGKLPVPWPTLVTTLTYGVQCFSRYPDHMKQHDFFKSAMPEGYVQERTIFF
KDDGNYKTRAEVKFEGDTLVNRIELKGIDFKEDGNILGHKLEYNYNSHNVYIMADKQKN
GIKVNFKIRHNIEDGSVQLADHYQQNIPIGDGPVLLPDNHYLSYQSALSKDPNEKRDHM
VLLEFVTAAGITLGMDELYKRGSASGGGGGLVPRGSASGAVSELIKENMHMKLYMEG
TVNNHHFKCTSEGEGKPYEGTQTMRIKVVEGGPLPFAFDILATSFMYGSYTFINHTQGI
PDFFKQSFPEGFTWERVTTYEDGGVLTATQDTSLQDGCLIYNVKIRGVNFTSNGPVM

QKKTLGWEAGTEMLYPADGGLEGRSDDALKLVGGGHLICNLKSTYRSKKPAKNLKVP
GVYYVDRRLERIKEADKETYVEQHEVAVARYCDLPSKLGHRGKDEL

Nucleotide sequence of LSSmsfClopHensor:

CACCATGGTGAGCAAGGGCGAGGAGCTGTTCACCGGGGTGGTGCCCATCCTGGT
CGAGCTGGACGGCGACGTAAACGGCCACAAGTTCAGCGTGCGCGGCGAGGGCG
AGGGCGATGCCACCAACGGCAAGCTGACCCTGAAGTTCATCTGCACCACCGGCAA
GCTGCCCGTGCCCTGGCCCACCCTCGTGACCACCCTGACCTACGGCGTGCAGTG
CTTCAGCCGCTACCCCGACCACATGAAGCGCCACGACTTCTTCAAGTCCGCCATG
CCCGAAGGCTACGTCCAGGAGCGCACCATCAGCTTCAAGGACGACGGCACCTAC
AAGACCCGCGCCGAGGTGAAGTTCGAGGGCGACACCCTGGTGAACCGCATCGAG
CTGAAGGGCATCGACTTCAAGGAGGACGGCAACATCCTGGGGCACAAGCTGGAG
TACAACTTCAACAGCCACAACGTCTATATCACCGCCGACAAGCAGAAGAACGGCAT
CAAGGCCAACTTCAAGATCCGCCACAACGTGGAGGACGGCAGCGTGCAGCTCGC
CGACCACTACCAGCAGAACACCCCCATCGGCGACGGCCCCGTGCTGCTGCCCGA
CAACCACTACCTGAGCTACCAGTCCGTGCTGAGCAAAGACCCCAACGAGAAGCGC
GATCACATGGTCCTGCTGGAGTTCGTGACCGCCGCCGGGATCACTCACGGCATGG
ACGAGCTGTACAAGCGCGCGTCTGGTGGTGGTGGTGGTCTAGTTCCACGTGGATC
TGCCTCAGGAGCAGTGAGCGAGCTGATTAAGGAGAACATGCACATGAAGCTGTAC
ATGGAAGGCACCGTGAACAACCACCACTTCAAGTGCACATCCGAGGGCGAAGGCA
AGCCCTACGAGGGCACCCAGACCATGAGAATCAAGGTGGTCGAGGGCGGCCCTC
TACCCTTCGCCTTCGACATCTTGGCTACCAGCTTCATGTACGGCAGCTACACCTTC
ATCAACCACACCCAGGGCATCCCCGACTTCTTTAAGCAGTCCTTCCCTGAGGGCTT
CACATGGGAGAGAGTCACCACATACGAAGACGGGGGCGTGCTGACCGCTACCCA
GGACACCAGCCTCCAGGACGGTTGCCTCATCTACAACGTCAAGATCAGAGGGGTG
AACTTCACATCCAACGGCCCTGTGATGCAGAAGAAAACACTCGGCTGGGAGGCCG
GCACCGAGATGCTGTACCCCGCTGACGGCGGCCTGGAAGGCAGATCTGACGACG
CCCTGAAGCTCGTGGGCGGGGGCCACCTGATCTGCAACTTGAAGAGCACATACAG
ATCCAAGAAACCCGCTAAGAATCTCAAGGTGCCCGGCGTCTACTATGTGGACCGA
AGACTGGAAAGAATCAAGGAGGCCGACAAAGAGACCTACGTCGAGCAGCACGAG
GTGGCTGTGGCCAGATACTGCGACCTCCCTAGCAAACTGGGGCACCGCTAA

Amino acid sequence of LSSmsfClopHensor:

MVSKGEELFTGVVPILVELDGDVNGHKFSVSGEGEGDATYGKLTLKFICTTGKLPVPW
PTLVTTLTYGVQCFSRYPDHMKQHDFFKSAMPEGYVQERTIFFKDDGNYKTRAEVKF
EGDTLVNRIELKGIDFKEDGNILGHKLEYNYNSHNVYIMADKQKNGIKVNFKIRHNIEDG
SVQLADHYQQNIPIGDGPVLLPDNHYLSYQSALSKDPNEKRDHMVLLEFVTAAGITLG
MDELYKRGSASGGGGGLVPRGSASGAVSELIKENMHMKLYMEGTVNNHHFKCTSEG
EGKPYEGTQTMRIKVVEGGPLPFAFDILATSFMYGSYTFINHTQGIPDFFKQSFPEGFT
WERVTTYEDGGVLTATQDTSLQDGCLIYNVKIRGVNFTSNGPVMQKKTLGWEAGTEM
LYPADGGLEGRSDDALKLVGGGHLICNLKSTYRSKKPAKNLKVPGVYYVDRRLERIKE
ADKETYVEQHEVAVARYCDLPSKLGHR

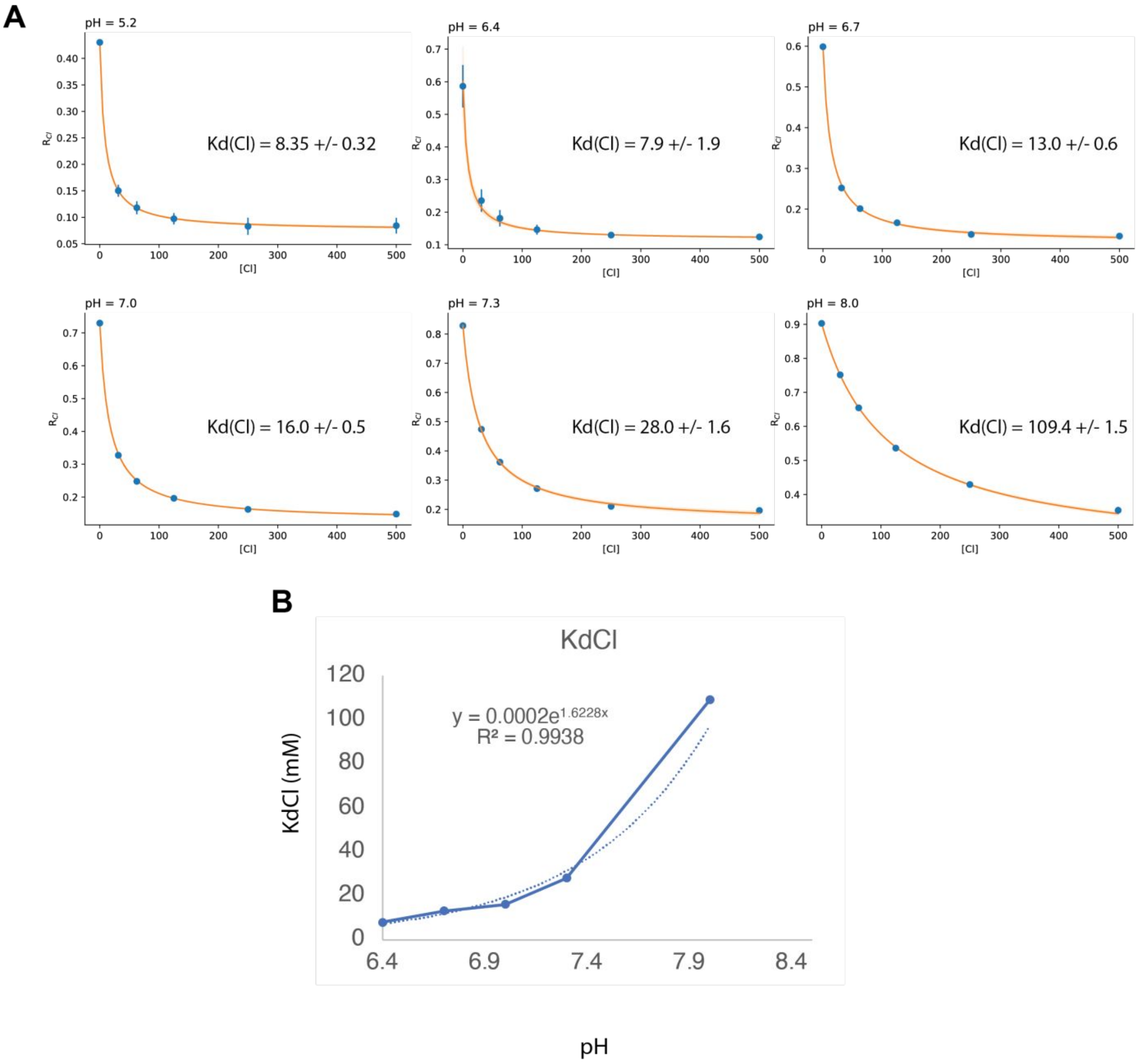

Supplemental Figure 2: KdCl versus pH. (A) KdCl of recombinant LSSmsfClopHensor was determined at the indicated pH by varying chloride concentrations from 0 to 100 mM (top center plot is a repeat of Figure 1F shown for comparison). **(B)** The best fit exponential function is shown and was used to calculate KdCl for the measured pH for eventual determination of [Cl-].

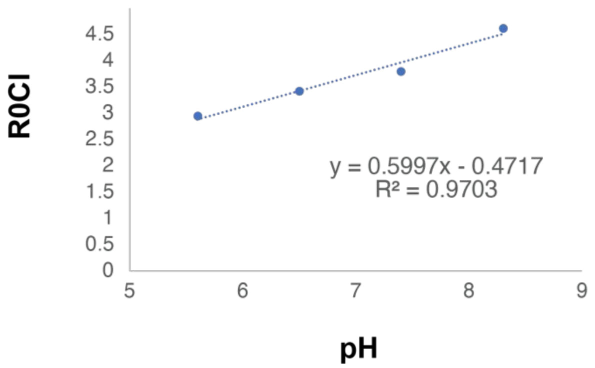

Supplemental Figure 3: R0Cl dependence on pH. The RCl values at 0 mM [Cl-] (R0Cl) are plotted versus the pH from the in-cell calibration (Figure 3D). The fit curve used to estimate R0Cl at any given pH is shown.

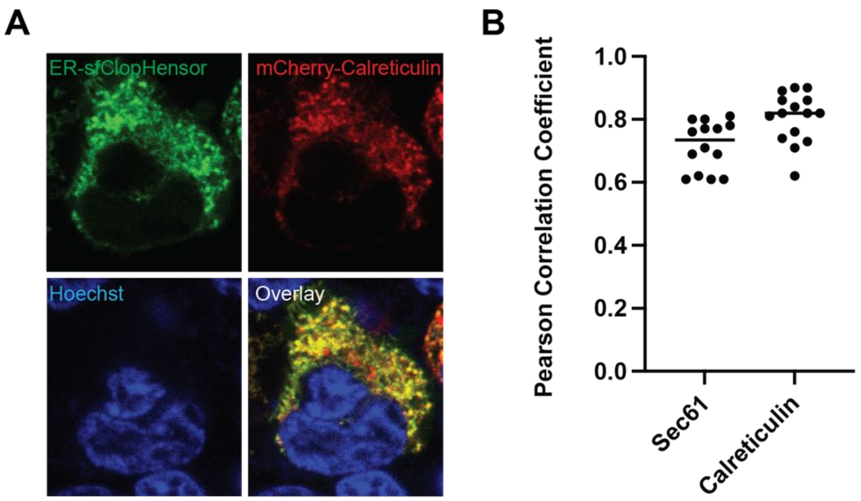

Supplemental Figure 4: Colocalization of ER-LSSmsfClopHensor with ER markers. 293T cells were transected with ER-LSSmsfClopHensor and mCherry-calreticulin. **(A)** 48 hours after transfection cells were imaged live and imaged for GFP (upper left), mCherry (upper right), or Hoechst (lower left). **(B)** Pearson correlation coefficients between GFP and mCherry for the indicated ER marker (fused to mCherry). Each dot represents a cell.

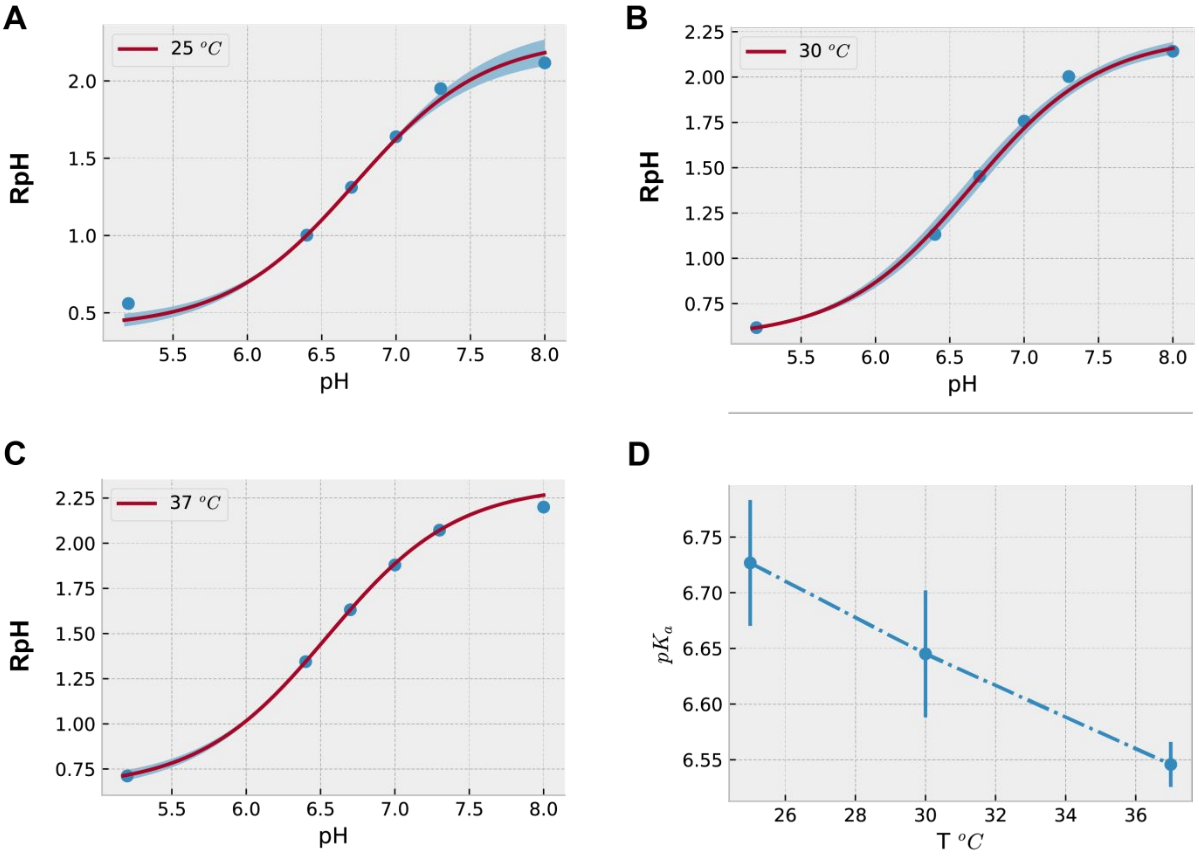

Supplemental Figure 5: Dependence of RpH and pKa of LSSmsfClopHensor on temperature. Recombinant LSSmsfClopHensor was placed in buffer of the indicated pH at 0 mM [Cl-]. RpH was calculated as green / cyan and best fit to calculate RA, RB and pKa. **(A)** at 25 degrees C **(B)** at 30 degrees C **(C)** at 37 degrees C. n=2 replicates per condition. Blue shading around the red trend line indicates the 95% confidence interval. **(D)** Plot of pKa versus temperature. Error bars indicate standard deviation of the best fit line.

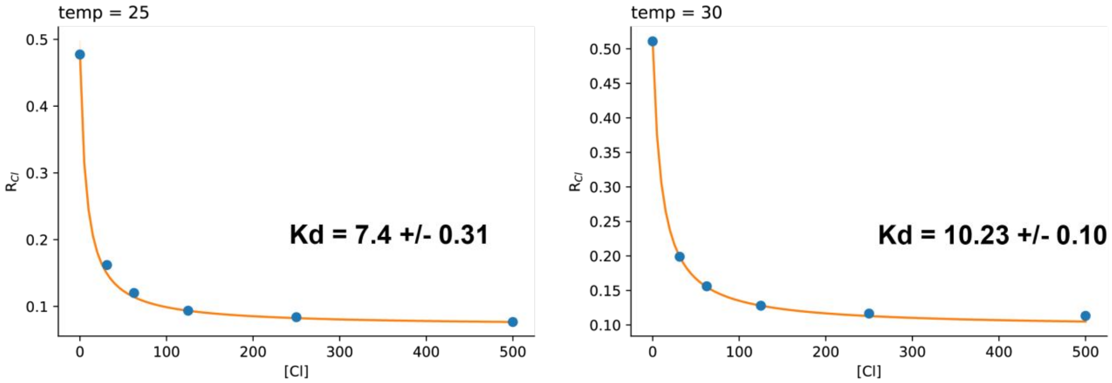

Supplemental Figure 6: Dependence of RCl and KdCl on temperature. As in Figure 1F, but taken at the indicated temperature. Best fit KdCl is indicated. n=2 replicates for each condition.

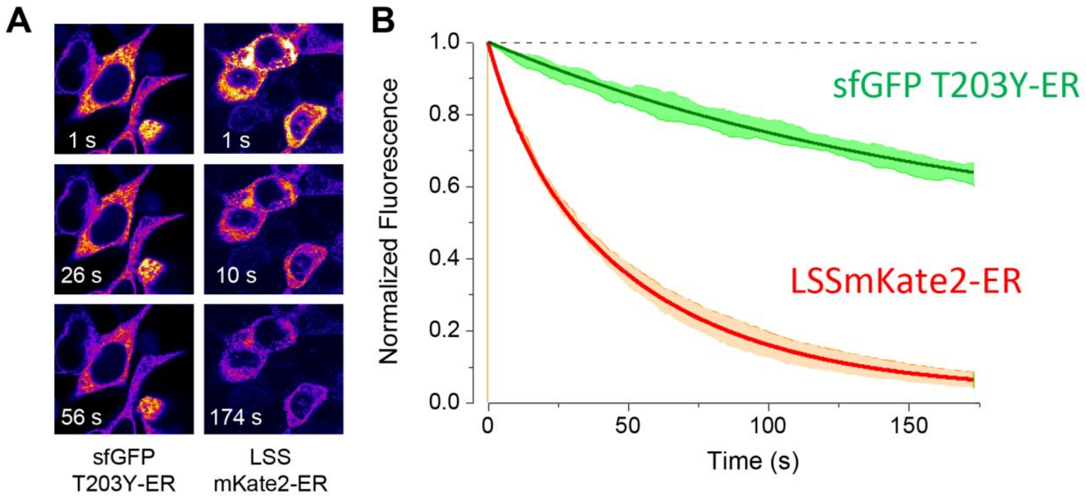

Supplemental Figure 7: Photobleaching of sfGFP T203Y-ER and LSSm-Kate2-ER. 293T cells were transfected with the indicated plasmid and imaged every second for 175 seconds. **(A)** Representative images at the indicated time. **(B)** Plot of the normalized fluorescnece. Double exponential fit line for LSSmKate2-ER and single exponential fit line for sfGFP is shown.

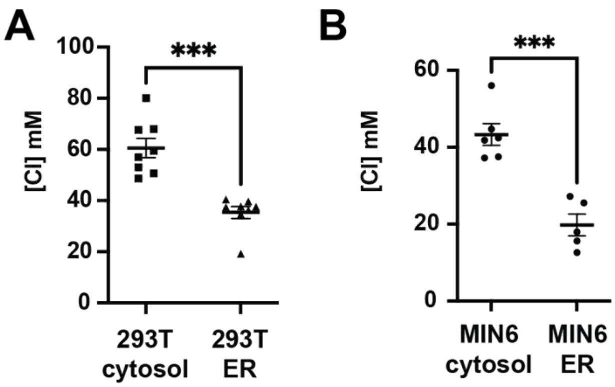

Supplemental Figure 8: Non-pH corrected estimated [Cl]. The data from Figure 5A and C was plotted assuming a pH of 7.4, R0Cl 4, and Kd of 39 mM. **(A)** 293T cells **(B)** MIN6 cells. ***p<0.001 by Welch's t-test.

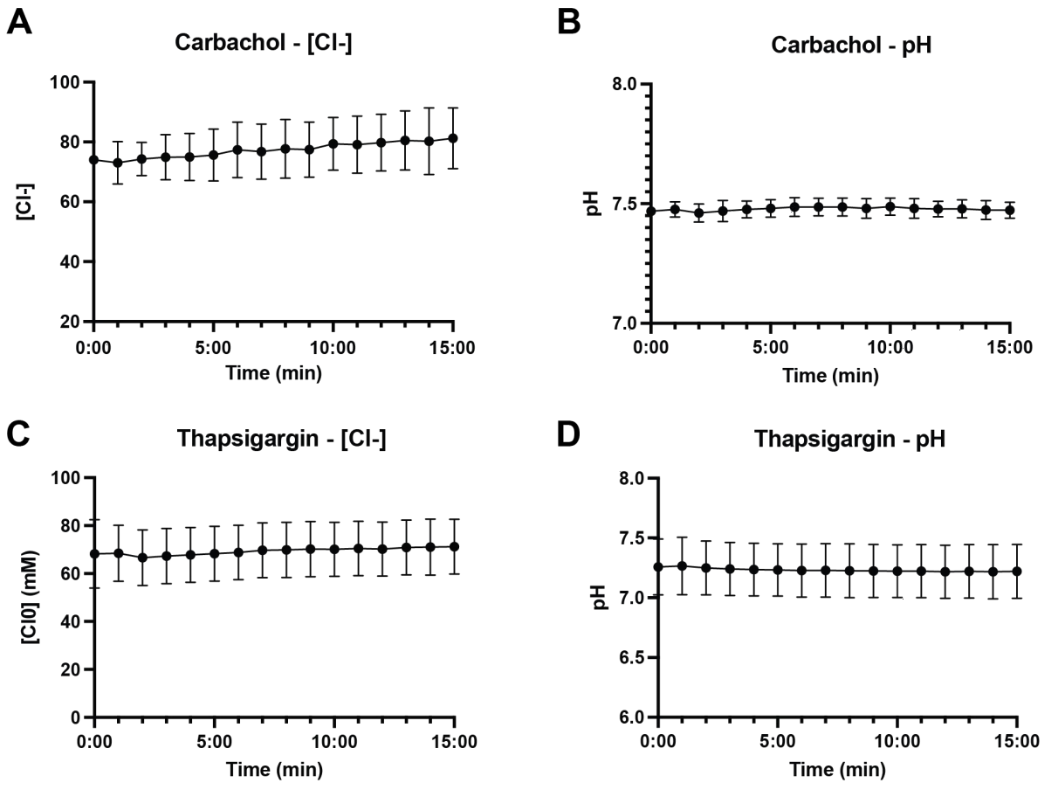

Supplemental Figure 9: Carbachol or thapsigargin do not measurably alter ER [Cl-]. 293T cells were transiently transfected with ER-LSSmsfClo-pHensor. 48 hours after transfection, cells were equilibrated in HBSS with 50 mM HEPES pH 7.0 and 450 mg/dL glucose for 1 hour at 37 degrees. **(A)** 100 uM carbachol was added at time zero and an image was taken every 1 minute for 15 minutes. Calculated chloride is plotted. **(B)** As in A but calculated pH is plotted. **(C)** 10 uM thapsigargin was added at time 0. Calculated chloride is plotted. **(D)** As in C but calculated pH is plotted. n=3 fields of cells for carbachol, n=5 for thapsigargin, error bars show standard error.
